# #Skinny girls: young girls’ learning processes and health-related social media

**DOI:** 10.1080/2159676X.2021.1888152

**Published:** 2021-03-03

**Authors:** Victoria Goodyear, Joacim Andersson, Mikael Quennerstedt, Valeria Varea

**Affiliations:** a School of Sport, Exercise and Rehabilitation Sciences, University of Birmingham, Birmingham, United Kingdom of Great Britain and Northern Ireland; b School of Health and Medical Sciences, Orebro University, Orebro, Sweden

**Keywords:** Education, body image, physical activity, diet, adolescence, instagram

## Abstract

This paper provides in-depth knowledge into young girls’ learning processes in relation to physical activity, diet/nutrition and body image. Data were generated from interviews with 49 girls (age 13–15) in England. The practical epistemological analysis technique was used to explore young people as both producers and consumers, or prosumers, of content and knowledge. The data illustrate that adolescent girls navigate two interrelated health-related paradoxes within publicly private spaces: (i) skinny fat and (ii) naturally fake. Skinny fat refers to how participation in social media represents a continuous struggle of becoming skinny, but at the same time not trying too hard to become too skinny. Naturally fake refers to how having a ‘natural’ look is highly valued, but equally, it is acceptable to be ‘fake’. Overall, adolescent girls are competent users of social media, who are able to navigate the complexity of the medium and its contents. At the same time, the adolescent girls sometimes found themselves, unintentionally, exposed to risks (e.g. bullying or body dysmorphia), particularly when social media was experienced publicly in a temporal order, connected to the past or present, and without control of potential future effects and impacts. Relevant adults should acknowledge young people’s vast competence of life on social media and further empower young people to self-regulate their learning through social media, and in ways that help them to learn from experiences about their health and bodies to shape future actions.

## Introduction

Social media is integral to young people’s lives, and is thus significant in relation to their education and health (Askari et al. 2018; Greenhow and Lewin 2016). For example, 82% of young people (age 12–15) in the UK use social media to access information, socialise, and for entertainment and emotional support (Brooks et al. 2020). Hence, social media is more than a tool that young people use. Social media is a powerful enabler that informs habits, and influences attitudes, knowledge and behaviours (Third et al. 2019). Consequently, many adults who have a responsibility for young people (including parents/carers, teachers, policy makers, and health professionals) are ambivalent about social media (Livingstone and Third 2017). On the one hand, evidence suggests that social media is an optimal medium through which to reach young people (including those who may be vulnerable) to communicate health messages and develop positive health behaviours (Freeman, Caldwell, and Scott 2020; Shaw et al. 2015), an opportunity acknowledged by the World Health Organisation in the Global Physical Activity Action Plan World Health Organization [WHO] 2018). At the same time, there are certain health-related harms that might result from social media, such as addictive behaviours, cyberbullying or accessing/sharing inappropriate content (Third et al. 2017), an aspect which UNICEF have cautioned (Kardefelt-Winther 2017). The key challenge for relevant adults therefore is not to dismiss, ban or block social media, but to support young people to engage with social media safely, responsibly, and effectively. Hence, leading regulatory bodies across the world have signalled the urgent need for new evidence to explain and support relationships between young people, social media and health. For example, a report by the Science and Technology Parliamentary Committee in the UK identified that a key priority area for new research is generating evidence on the nature of adolescents’ health-related interactions and experiences on social media (House of Commons 2019).

Our current knowledge on the relationship between young people, social media and health is predominantly based on research that attempts to quantify social media use (i.e. time) and examine the association with, for example, physical activity levels, sleep duration, and/or mental health outcomes (e.g. Viner et al. 2019; Scott, Biello, and Woods 2019). Although this has been an important first step, the current evidence is insufficient. We need to know more about young people’s investments in social media as part of their lives, and the chaotic, dynamic and ongoing ways in which young people consume and produce content (Third et al. 2019; Toffoletti et al. 2021; Toll and Norman 2021). Notably, systematic reviews on social media interventions for physical activity, diet and wellbeing for adolescents have been published, but none have provided robust evidence that identifies the characteristics of social media use that are associated with positive physical activity, diet and wellbeing outcomes (c.f. Chau, Burgermaster, and Mamykina 2018; Hamm et al. 2014; Hsu et al. 2018). Hence, little is known about the types of social media activities that inform, develop or shape young people’s health-related attitudes, knowledge and behaviours (Camacho-Miñano, MacIsaac, and Rich 2019; Dickinson et al. 2019; House of Commons 2019; Scott, Biello, and Woods 2019).

This paper provides in-depth knowledge into the learning going on through the dynamic ways in which young people use social media in relation to health, with a specific focus on physical activity, diet/nutrition and body image. The focus is on what is sometimes referred to as the ‘black box’ of learning in the relationship between young people, social media use and health, i.e. between input and output. In this paper, we examine adolescent girls’ (age 14–15) learning via health-related social media. Building on our earlier work (Goodyear and Armour 2019), as well as others (Booker, Kelly, and Sacker 2018; Kelly et al. 2019), we have identified specific gender differences in the ways in which adolescent boys and girls use social media. Yet, there are very few gender-specific analysis of social media use (Camacho-Miñano, MacIsaac, and Rich 2019; Booker, Kelly, and Sacker 2018; Toffoletti et al. 2021), and even fewer studies that foreground young people’s voices in the research (Third et al. 2019). Our analysis is thus centred on the perspectives and experiences of adolescent girls’ engagement with health-related social media, and how ways of learning influence the route learning takes. The research question guiding this study was: What social media activities do adolescent girls engage with that informs their health-related attitudes, knowledge and behaviours? Accordingly, in this paper we identify the mechanisms that shape, inform and/or develop girls’ health-related attitudes, knowledge and behaviours. This evidence can be used to inform effective policy and practice to harness the health and educative potential of social media, and mitigate any potential risks.

## Young people, social media and learning: theoretical framework and methodological considerations

To investigate how ways of learning influence the route learning takes and describe adolescent girls’ functional coordination of health experiences on social media, we use a methodological approach called Practical Epistemological Analysis (PEA). PEA has proved valuable for empirical analysis of learning processes and the direction of learning in connection to health-related interactions and experiences on social media (e.g. Goodyear and Quennerstedt 2020; Carlén and Maivorsdotter 2017). In the context of this paper, PEA provided an analytical tool to: (a) explore young people as both producers and consumers, or prosumers, of content and knowledge; (b) open up, rather than close down, ways of theorising young people’s uses of social media; and (c) explore the intricate details on how specific aspects of social media are used.

Overall, PEA is a technique for analysing how embodied experiences are structured during specific encounters, and is based on the works of Wittgenstein and Dewey. In Dewey’s transactional perspective (Dewey and Bentley [1949] 1991), knowledge is not exclusively located in the minds of humans, but is something practical that people do when acting in the world. Accordingly, the meaning of something (e.g. a word or image, or an artefact) can be found in its use, and when, for example, a certain way to talk or act in a shared context is privileged the participants can be understood as active co-creators of certain *practical epistemologies* (Andersson, Garrison, and Östman 2018; Wickman and Östman 2002). From this perspective, the meaning of social media for health in adolescents lies in the transactional engagement and experiences of adolescents’ actual uses of social media (i.e. what they produce). Building on Dewey (1929), experience refers to the different ways in which transactions take form in a specific situation and includes the experiencer as well as what is experienced. To learn from experience accordingly refers to connecting the past – i.e. previous experiences – with future – i.e. the direction of experiences (Dewey 1916). Yet, experience is not merely private but extends simultaneously into the public view (Andersson and Garrison 2016). This means that individuals can experience (consume) different things individually but still share the same situation.

Adolescents’ engagement with social media, from a PEA perspective, is accordingly a continuous functional coordination between actions (producing) and experiences (consuming), social media content, the materiality of the device, or other participants on social media. By using the analytical concepts; purpose, gaps, relations, stand fast and encounters, Wickman and Östman (2002, p.603) argue that learning in this way can be studied as “a process where gaps are filled by constructing difference and similarities in relation to what is immediately intelligible”. For the purpose of this paper, PEA works as a methodological technique for analysing how adolescent girls’ experiences are coordinated during specific ‘encounters’ with physical activity, diet/nutrition and body image, how they rely on what is certain and given (what ‘stands fast’), how they try to navigate disrupted situations and meanings (‘gaps’) and how they make judgements and acquire habits (‘relations’).

## An overview of the methods

### Context

The participants and data reported in this paper is part of a wider participatory action research project examining young people’s uses of health-related social media, and from the perspectives of young people (see Goodyear and Armour 2019). In the main project, data were collected from 1346 young people (age 13–18) and was carried out in 10 UK schools, and across 12 classes (4 mixed gender, 3 boys, and 5 girl only classes). The schools were representative of different school types in the UK,^1^ where of the 10 schools, 2 were private, 3 were state and 5 were academies, 2 of which were faith schools. Furthermore, the schools were located in diverse socio-economic areas and included students from a range of Black, Asian and Minority Ethnic groups. In the research project, social media was used by the majority of the young people (99%), and was defined broadly by the young people to include social networking (e.g. Instagram, Facebook, SnapChat), media sharing (e.g. YouTube), discussion networks (e.g. WhatsApp), and bookmarking (e.g. Pinterest) forms of social media. From this larger project we can also conclude that young people were critical prosumers of social media and its contents. For example, over half of the young people in the study actively looked for health information on social media (53%) to inform their knowledge, skills and behaviours related to physical activity, diet and body image, and many of the young people reported that they would swipe past content if it was deemed irrelevant to their bodies (57%). For further details about the wider project please see Goodyear and Armour (2019).

One key objective of the wider project was to identify the learning mechanisms that shape, inform and/or develop young people’s health-related attitudes, knowledge and behaviours. In this paper we report on the findings related to that objective. In the process of analysis we identified two segments in the data – skinny girls and gymlads – that emphasised gender-specific uses of social media, and gender-specific learning mechanisms. To represent these social media behaviours in depth, the first paper reported on gymlads, as being about young boys engagement with social media-based gym culture involving images and videos of workouts (see Goodyear and Quennerstedt 2020). In this second paper we report on skinny girls, as being about how young girls present their bodies on social media. Hence in this section we provide an overview of the data collection techniques and analytical process.

#### Ethics

Overall a culturally responsive relational and reflexive approach to ethics was adopted (Goodyear and Armour 2019; Sparkes and Smith, 2014). This meant that ethical decision-making was contextualised in relation to young people’s autonomy in digital spaces and their digital rights, with respect for privacy. University ethical approval was granted and informed consent or assent were obtained.

### Data collection

The sample for this paper was determined post data collection, and when gender-specific uses of social media use were identified from thematic analysis of the data generated across the wider project (see Goodyear and Armour 2019). The sample includes data that were collected from 49 young girls (age 13–15) who participated in 15 Focus Group (FG) interviews. The FGs took place with the girls in their schools, and included girls from the same class. Of the 15 FGs, data were generated from 7 of the 10 schools in the wider sample (that included private, state, and academy schools, including a faith school). The majority of the FGs were single sex (n = 11), and where data were collected from mixed-sex FGs the voices of boys were excluded from the analysis. The FGs were led by a researcher and the content and format of the FG interviews were informed by data previously collected from the girls as part of the wider project – such as data from class activities generated in the form of questionnaires, quizzes and digital pinboards on the types of social media used and images/videos accessed from social media in relation to physical activity, diet and body image. Elicitation techniques were used to encourage the girls to discuss their digital pinboards (for example, in looking at the digital pinboard, can you explain why you are more interested in social media posts shared by celebrities, like Kylie Jenner, rather than health organisations, like the National Health Service [NHS]). Semi-structured questions were then used to understand the girls’ experiences of social media, and were common across all groups (for example, when you post to social media about your health, can you explain the types of posts you share and why). The average duration of FGs was 38 minutes, and the FGs were voice recorded and later transcribed.

### Data analysis

Data analysis was guided by our objective to investigate the perspectives and experiences of adolescent girls’ engagement with health-related social media, and how ways of learning influence the route learning takes. PEA was employed as an appropriate analytical technique for this analysis as it engages with the complexity and multi-directionality in the relationships between adolescence, social media and health, rather than assuming linearity to the influence of e.g. time on social media or content, that has dominated most existing research (Dickinson et al. 2019). The analysis took place in two overarching phases.

In phase 1, as reported in Goodyear and Quennerstedt (2020), the interview transcripts were organised and read through, and this process led to the identification of the 2 segments of data – *gymlad* and *skinny girls*. In phase 2, the two categories of data were analysed separately using PEA (see Andersson, Garrison, and Östman 2018). Following on from established work that has used PEA, five concepts were used for in-depth analysis: (i) purpose; (ii) gaps; (iii) relations; (iv) stand fast and (v) encounters. These five steps are described in Table 1.

Using this approach, five analytical questions from the PEA concepts were developed and used by all co-authors first separately and then collectively to code and categorise the data:

- What is the purpose of young people’s engagement with health-related social media?
- What gaps are identified in young people’s experiences of health-related social media?
- What do young people do, use, or draw upon to ‘fill’ the gaps in attitudes, knowledge and/or behaviours?
- What is obvious or taken for granted for young people in relation to health-related social media?
- What issues or questions do they engage with in relation to the identified gaps?

By using these questions, the authors identified two distinct indeterminate situations in relation to how the girls are coordinating their experiences of health-related social media within the overarching data set (and these are discussed in the results section as a zoomed-out perspective). The author team then selected one interview transcript to analyse and illustrate these situations in further detail, using the 5 PEA concepts (discussed in the results section as zoomed in perspective). In the discussion, we further explore the indeterminate situations in relation to theory and previous literature.

#### Deliberation

In all phases of the research, a *deliberative strategy* was utilised in order to evaluate each step in the research process in terms of quality (Goodyear, Kerner and Quennerstedt 2019). In this process we were inspired by Tracy’s (2010) end goals for excellent qualitative research and Guba and Lincoln (2005) perspectives of authenticity in research.

A deliberative strategy involves ‘intelligent deliberation and balanced consideration of alternatives through mutual communication’ (Englund 2006 p.508). The goal is not to come to a singular truth, but to reach as high-quality research as possible using established criteria for high-quality research (Tracy 2010). The deliberative strategy involves all authors being given the possibility to make judgements in relation to different alternatives, views and arguments. Bringing together authors from different backgrounds and experiences is thus a respectful endeavour to achieve rich deliberation. The deliberative strategy in this study aimed to ensure the findings were sufficiently authentic in order to facilitate engagement and impact (Guba and Lincoln 2005).

Young people, social media and health is a worthy topic, and is currently receiving significant attention in policy, research, and practice fields (c.f. House of Commons 2019). In this context, academic research has been scrutinised for opportunistic research that pursues this topic in shallow ways and with little care devoted to design and data collection, that are representative of young people’s perspectives and experiences (Orben and Przybylski 2019). Through the deliberative strategy we, with inspiration from Guba and Lincoln (2005), sought to address: (i) ‘educative authenticity’ to raise stakeholder awareness of young people’s experiences in ways that would influence moral actions; (ii) fairness, through the illustration of data that provided an authentic representation of all 49 girls in the sample perspectives, and avoided researcher bias from the co-author who had led the overarching project (Guba and Lincoln 2005). The team for this paper were selected to provide different and broad disciplinary expertise that were relevant to the data set, and on: social media and social/emotional systems (Author 1 - Goodyear), learning (Author 3 - Quennerstedt), gender (Author 4 - Varea) and embodiment (Author 2 - Andersson), as they relate to young girls. Our joint expertise lies in (a) how we understand the wider context of social media communication and (b) our differential expertise in relation to education and health. The diverse author team and their differential engagement with the data is therefore a key resource informing authenticity and quality in this paper.

## Results

In the results section, a zoomed-out perspective and a zoomed-in empirical short story of the data is provided in relation to the five PEA concepts to illustrate the complexity of young girls’ uses of social media. As presented in Goodyear and Quennerstedt (2020) stories can provide dense descriptions and facilitate rich interpretations of key findings (Phoenix, Smith, and Sparkes 2010; Smith et al. 2013). The short story reported in this paper is derived from the data under a general theme of #*Skinnygirls* (see Goodyear and Quennerstedt 2020 for the presentation of the other theme *#Gymlad*). Accordingly, the stories are representative of the wider data set while at the same time providing an opportunity to present the results in more detail.

### Learning – a zoomed-out perspective

Social media is always present and taken-for-granted for the girls – everyone is on social media. The overall *purpose* of engaging in social media is to be ‘ordinary’, as being on social media is how life is. The girls *live* social media, as opposed to just *using* it:

Female: Everyone our age has that [a social media account], so that’s what we use to communicate.

Female: If they haven’t got it, it means they’re dead people.

Female: Yes, basically.

Participation in social media is driven by the need to be socially responsive, and this means that adolescent girls are always in a responding position to posts, likes, comments and stories. Unwritten rules exist in their participation in social media. For example, girls need to notify their followers when they are unavailable to respond. They will post to their story NITM [Not In The Mood] no replies”. The ongoing responsivity means that adolescent girls cannot easily dismiss or ignore the vast amounts of content that are accessible to them, and this also includes health-related content. To be socially responsive girls think through what they view, swipe away, like and post. Social media is thus an empirical real-life concern (e.g. testing apps [e.g. 7-minute workout app] and smoothies, getting motivated for physical activity by images, and suffering bullying) where the norm is to consume and produce authentic content, and where validity is socially determined through evaluations of ongoing stories and responses (i.e likes, comments, views). As such, while many adolescent girls do not explicitly associate social media with health, health is a significant part of their experiences of social media and learning.

To live, social media for adolescent girls means to always be publicly exposed and therefore potentially at risk. One example of how girls are regulating their experiences of this publicity is seen in how they are capable of making distinctions between a public personal domain and a public-private domain, which involves different degrees of intimacy and appropriacy. Using a more private publicity – such as in closed groups of WhatsApp – messages and images seem to have more validity because they are seen as more intimate. Content posted in the public personal domain – e.g. through stories that are ephemeral and share temporary moments – are often regarded as inappropriate or just uninteresting. To know how to balance the public personal and private-public involves both judging what might be intimate, appropriate or interesting for others; and also knowing how the responding feeds and comments will link up to other posts. Producing public personal content is consequently associated with risks in that it can turn into public-private content depending on what 'likes' it got once posted.

Overall, our analysis reveals that these adolescent girls navigate two interrelated health-related paradoxes within publicly private spaces: (i) *skinny fat* and (ii) *naturally fake*. The two paradoxes represent distinct indeterminate situations in relation to how the girls are coordinating their experiences of health-related social media in a general sense.

*Skinny Fat*: Participation in social media represents a continuous struggle of becoming skinny, but at the same time not trying too hard to become too skinny. Health accordingly becomes about looking good, being fit and being skinny – #skinnygirls – but at the same time preferably also having an hour glass shaped body within a quite heterosexual norm.

Female: Because if you are skinny, they will look at you. If you are too fat, they will look at you, they will say something about it. Everyone just has to be slim thick^2^ basically.

Female: On Instagram, you’ve got to be either thick with a big bum or something, or you have to be like really skinny with a flat stomach.

Being fat or overweight is constructed as the opposite of being fit, and involves constant progression, always transforming, always becoming better, and constantly shaping the body. Health for adolescent girls is thus something visible, the body’s appearance. Yet presenting yourself as too ‘healthy’ or in a way that is admirable – such as skinny-fat or slim-thick – can cause anxiety in other adolescent girls, through implicitly ‘fatshaming’^3^ other adolescent girls into being skinny, understood as more normal.

*Naturally Fake*: Having a ‘natural’ look is highly valued to adolescent girls, particularly in terms of how they present themselves on social media. Equally, it is acceptable to be ‘fake’, but as long as this is not too extreme, not too ‘fake’. The boundary that establishes ‘fake’ versus natural bodies is therefore complex and unclear. Adolescent girls navigate the boundary of naturally fake through their evaluations of visual images (e.g. celebrities and peers their age), and associated comments and likes. They feel a level of genuine pressure when images are of people their own age, rather than celebrities with unattainable ideals (e.g. doing surgery or celebrities like the Kardashians). Yet the comments and likes that accompany images disrupt authenticity and can lead to frustration and anger rather than admiration and inspiration. For example, some adolescent girls use ‘fat talk’ to accompany their images – that is, slim girls commenting on how fat they are.

Female: I feel like when people our age that we know post photos of them and their body and they know …

Female: … they know that it’s better than others, and then the caption will be like “chunky monkey” or something.

Female: Saying bad stuff about their figure, when they clearly know that their figure can’t be that bad otherwise they wouldn’t have posted the picture in the first place.

Female: When their figure is better than mine and they’re saying that they’re fat …

Female: Then you think that yours is 10 times worse.

Female: I don’t like that.

Other adolescent girls provide ‘fake’ compliments, in the form of likes and comments (e.g. „I wish I could look like that”) when the natural bodies are not ‘good enough’. The dismantling of fake on social media is thus about determining who is more or less ‘natural’.

### Learning – a zoomed-in perspective

In the zoomed-in perspective we extrapolate details of how the adolescent girls coordinate embodied experiences according to their more situated purposes of their lives on social media in relation to the two paradoxes: *skinny fat* and *naturally fake*. The data is taken from one interview with the same group of young girls. This helps to explain what gaps the girls are experiencing, what encounters they are making with shaming/fat talks and practices, how they are trying to bridge theses gaps by creating certain relations and what stands fast for them in this navigating of health on social media.

#### Skinny fat

The *purpose* of engagement with health-related social media is to access and follow visual stories of their friends’ and celebrities’ everyday lives.

1. Moderator: What posts do you look at on there?

2. Female: Selfies.

3. Moderator: Selfies?

4. Female: Yeah, other people’s stories …

5. Moderator: Other people’s stories, okay. And so do you look at your friends?

6. All: Yeah.

7. Moderator: Or celebrities?

8. Female: Yeah.

9. Female: Both.

10. Moderator: Okay. But so you know that, so you know how to be healthy and how to do exercise and what to eat, but do you think other people with these images, are they a good or bad thing for other people?

11. Female: Probably for some …

12. Female: It depends on how you think about things.

These stories^4^ are in order to make sense of what it means to be ‘ordinary’, normal and/or to become healthy (L1-12), and such consumption of visual images leads to the identification of various *gaps*.

13. Female: For some people they intimidate …

14. Female: Yeah. And are like discouraged …

15. Female: It’s either like a goal of yours, or like it’s something that you don’t want to see.

16. Female: It’s almost like you feel like it’s fat shaming you – like saying you 'Have to look like this' …

17. Female: Some people are insecure about their bodies so …

18. Moderator: What does “fat shaming” mean?

19. Female: When someone says to you, „You shouldn’t be fat. You shouldn’t have … ” And stuff like that. Or, Some people are actually fat … ” And stuff like that. I think they get loads of people saying they shouldn’t be fat and things like that …

20. Moderator: Okay. So do people post the other way around? Could you have thin shaming”? …

21. Female: That’s actually a thing that doesn’t get recognised … Like I remember seeing this video and this girl, she got fat shamed and skinny shamed, because she was really fat and she lost loads of weight. And I think she kind of took it a bit too far – she was a healthy weight and then she took it a bit too far and so people started skinny shaming her.

22. Moderator: And did that happen on social media?

23. Female: Yeah.

24. Female: It happens a lot.

25. Moderator: What do you mean, how does it happen? Like people posting comments or like …

26. Female: Yeah, mostly comments.

In connection to the *purpose* of accessing and following visual stories, the girls are *encountering* different body-shame talks (L19, L21), as a quite accepted practice of ‘fat shaming’ or ‘skinny shaming’, that occur from celebrity and peer/friends’ posts (L1-11). Here the girls experience *gaps* between how others are exposing their bodies and talking about bodies and the way the girls perceive their own body. A *gap* also exists in the challenge of having a figure that is not fat (L16, L19) and at the same time not too skinny (L21, L29). In the continuing discussion the girls are *encountering* body-shame talks not only passively, when fat/skinny shaming involves an individual perceiving that a post is targeted at them, even though it may not be, and that the post is telling them to change their body (L16), but also on an active and personal level.

27. Female: people will start saying things like, “Oh you’re anorexic … ” And then …

28. Female: Yeah, I used to be really skinny – well, skinnier than this – and everyone used to be like, “Oh, you’re too skinny, you need to eat more„. I eat a lot, like a lot a lot. But like it’s not like something you can really help.

29. Female: Yeah, there are a lot of guys in my year, who because they’ve got such a fast metabolism, like I know … Like Mia she eats more than I do, but because my metabolism is like really fast, she’ll like just … She’s really skinny.

30. Moderator: It seems a bit harsh though to thin shame or fat shame, do you think? That’s a bit like – you must have done all the cyber bullying and stuff in school – do you think it’s a bit like that? …

31. Female: Yeah, it’s definitely like that.

32. Moderator: Do you think it’s worse than … Is it cyber bullying?

33. Female: Yeah, it is cyber bullying …

34. Female: I think it is.

35. Female: It’s saying you shouldn’t be a certain way.

36. Female: It’s worse because then people don’t feel good about themselves then …

37. Female: Yeah, it kind of discourages. And it kind of leads to some stuff that can be linked to like …

38. Female: Depression.

Through this active and personal *encounter* with body-shame talk, where they see a post that tells them that they are too fat (L19) or too skinny (L21, L29), the *gap* becomes reinforced and gaps between (i) between being skinny but not anorexic (L27) (iii) between eating and becoming skinny (L28-L29) also occur. These gaps between how their body responds to training and diet and how others want bodies to look like becomes particularly apparent when there are differences in rates of metabolism between adolescent girls (L28-L29). In the active and personal *encounter* with body-shame talk they create *relations* between skinny/fat shaming and bullying and depression. Furthermore, what *stands fast* is that health becomes a demand of a constant progression towards a certain body, and where health equals going to the gym, doing workouts, fitness and eating correctly. This becomes evident through the concept of *stand fast* and in how the *gaps* that lead to the girls feeling intimidated (L13), discouraged (L37) and/or depressed (L38). Moreover, the understanding that the body does not respond to training and diet in a “healthy” way, are filled with *relations* in terms of setting goals to change their bodies or something they avoid since perfect bodies are unrealistic (L15). This analysis illustrates the complex ways in which girls’ experience social media in relation to their health, and how social media activities inform their attitudes and knowledge related to the body.

In navigating the publicly private/personal spaces of social media, the girls also engaged in fat talk. In the last part of the transcript the girls begin to demonstrate irony in their uses of social media when they engage with fat talk.

39. Moderator: So does it happen – so you see these images – so does someone ever like post an image to get attention? You know like so they’ll be like they’re really thin, and then they’re saying, „;Oh, I’m so fat … ” Or something like that?

40. Female: Oh my god …

41. Female: You used to do that all the time …

42. Female: I don’t do that …

43. Female: You used to do that.

44. Female: I was like, “My thighs are big, damn it … ” And that’s all I’ve ever said.

45. Female: She used to say it all the time. Like skinny girls … Like I know skinny girls who are my friends and they say, „Oh I’m so fat, I’m so fat … ” And they’re literally like super skinny. And it’s like … Not in year eight … You used to be like in tears like, #x201C;I’m so fat … ”

46. Female: I did not, I did not.

47. Female: We used to say our thighs were big, and that was about it.

48. Female: No you … You’d like stick out your pelvis and go like, “I’m so fat … ” It’s like, „No … ”

49. Female: Okay, well that was year eight, year. Anyway …

Fat talk here involves pretending to be fat or have big thighs or to ironically portray themselves as skinny in order to gain positive feedback on their bodies (L39-49). This fat talk actually has the opposite effect (L45) and potentially leads to re-enforcing the three *gaps* above. A fit body is accordingly not necessarily a healthy body, but a body that fits a certain story, balancing body appearance between privately public and publicly private.

#### Naturally fake

The *purpose* of engagement with health-related social media for the adolescent girls is to communicate and present their bodies in certain ways through visual representations to their friends (L1-8). Not only do the girls have to judge what are appropriate comments to their own body, they are also dealing with the risk of posting the “wrong body”.

1. Moderator: Filters – are they really important?

2. Female: Yeah, because you can get like dog filters and then like pretty flower filters and funny filters.

3. Female: if it’s really important.

4. Female: Oh yeah, it is because if you’re having like a bad face day, you can put a filter over it. (Laughs)

5. Moderator: If you’re having a bad face day, okay. So you take selfies, then?

6. Female: I don’t really take selfies, but I send like a little photo to my friends, like if you’re messaging on SnapChat or something.

7. Female: I might take a picture with me and my friend with a filter, but then that’s it. I don’t take a selfie. (Laughs)

8. Moderator: Are selfies not a good thing?

9. Female: I mean, it is, but you have to … If you take one and you just don’t like it …

10. Female: Don’t post it.

In relation to this purpose, *gaps* occur between posts and their own aspirations what to present and they constantly navigate what is natural and what is fake in relations to what they encounter. The girls follow friends and celebrities and as in L1-10 post visual images not seldom modified by filters (L7-10). A *gap* can further be recognised in relation to what is deemed appropriate to post on social media. This gap is filled through posting images that use filters, and are obviously naturally fake (L1-7).

11. Female: They’re some YouTubers that you watch, they’re into fitness and everything. So they’ll post like healthy foods, and themselves with their gym body or something. And it’s not really like they’re saying that you should look like that, but kind of you think that.

12. Female: You’re thinking you should …

13. Moderator: So do you look at those posts?

14. Female: Yeah, because I like them. I like the people that do it.

15. Female: If I like the person, I might get motivated, but then …

16. Female: If I don’t, I just skip it.

17. Moderator: So a person your age, or a celebrity or a YouTuber?

18. Female: Like a celebrity or whatever.

19. Moderator: Like a celebrity. Okay. So can you give me an example …

20. Female: She’s not really a celebrity but she’s a YouTuber, she’s Naomi Smart.

21. Female: Kim Kardashian. She posts a lot of gym ones on SnapChat and everything.

22. Moderator: Okay, so when you see those posts, do they change any way you think or anything you do?

23. Female: No. I mean, when I see Kim Kardashian, you just think, no. She’s quite fake, to be honest.

24. Female: And she’s got … Like most of her isn’t real.

25. Female: Some YouTubers, they’re like healthy but the person I watch, she’s had no plastic surgery or anything. She just goes to the gym. It kind of makes me feel like motivated to do it, because it shows that you can without plastic surgery and that. But it doesn’t really bother me, because I just think, she’s an adult, she has her own life. But I’m a child. I’m at school still, so I don’t want to be like worrying about that.

26. Female: And all stressed and everything and that just adds to it then … .Well I mean, because when you see like … On Instagram, you’ve got to be either thick with a big bum or something, or you have to be like really skinny with a flat stomach. And if you’re in between it’s like, uhh.

27. Female: Which one are you going to be?

28. Female: Yeah.

29. Moderator: So there’s no real images, then, is that what you’re saying?

30. Female: Yeah.

Other gaps can be identified in relation to a *purpose* of following friends’ and celebrities’ visual images. Here the girls’ *encounters* are with: (i) celebrities and YouTubers, that present certain body ideals of how to look ‘healthy’ and (ii) ‘normal’ girls who either use photoshop or post their natural bodies without filters. *Gaps* exists between body images posted on social media and their own body (L11-16); celebrities and YouTubers (L18-25); and between natural bodies, trained gym bodies and fake gym bodies (i.e. photoshopped or modified through surgery) (L25-30). Gaps therefore exist between ideal bodies, and what is an appropriate concern for girls their age (L25), and feelings of stress (L26). A *relation* can be identified between being natural and thus healthy without plastic surgery and being motivated to do exercise (L15 L25). What *stands fast* is both (i) that visual representations on social media are the actual natural or fake representations of a person, and (ii) that natural equals health.

Further *gaps* exist between certain fake body ideals presented on social media and how much fake is acceptable, as well as between naturally fit bodies and extreme skinny or muscular bodies. This was apparent in the girls’ discussions of how filters and photoshop were used to modify body shape and size (L31-36).

31. Moderator: So some of the other girls said that they fake it almost, like they put a filter on.

32. Female: Or they Photoshop themselves and you can just see the round …

33. Female: Yeah, if they’re trying to put their butt in the photo or something but it’s like …

34. Female: You can see like the curve of it.

35. Female: You can see that they’ve Photoshopped it to make it look bigger.

36. Female: Yeah, and some people make their waists skinnier as well. Yeah. And then they smooth their face and everything … .

To fill these multiple gaps the girls created *relations* to fake and natural by determining what was ‘postable’ to social media and, in turn, what was motivating. They further filled the gaps with *relations* to natural bodies and fake and ‘photoshopped’ bodies where images without filters were considered motivational, whereas images that were obviously ‘photoshopped’, such as with really big bums, were ineffective motivational tools (L15, L37-43).

37. Female: I don’t think … We didn’t keep all of them, but I think we kept one because this girl looked natural. She didn’t look fake. She didn’t look too skinny or too muscly or something. We just said she looked nice.

38. Female: She looked natural.

39. Moderator: So natural is better?

40. Female: Yeah.

41. Moderator: So if you saw someone without a filter on them, is that more likely to … You’ll say, “Oh that’s real”?

42. Female: Yeah. If it’s like just a normal person – not a normal person, but a person that’s put their natural body on there without any filters or anything then you take that more into consideration.

43. Female: It’s not going to motivate me if I know that it’s not real by looking at the photos. Like say if someone posted a picture, they’ve Photoshopped it so they’ve got a really big bum and everything, that wouldn’t motivate me.

From analysing the Naturally Fake paradox what *stands fast* is that the visual representations on social media are the actual representations of a person. What also *stands fast* is that health is about eating habits, going to the gym, being fit and looking nice, where curves are admirable, and where you must exercise to be healthy. At the same time almost everybody fakes it on social media creating a boundary between what is acceptable and unacceptable as well as what naturally fake means.

## Discussion

This paper reports new evidence on the dynamic ways in which young girls use social media in relation to health as well as the mechanisms involved in adolescent girls learning about health and their bodies. Crucial to understanding these mechanisms is to acknowledge adolescent girls as highly competent participants in a complex social environment, and, that they continuously are balancing private and public aspect of their social media life.

Overall, our results illustrate that social media is intertwined with activities and practices that characterise adolescence, where the messiness and complexities of adolescent girls’ social lives, and social, biological and emotional development are part of everyday life that is lived through social media. Accordingly, in this data set there was evidence that adolescent girls are competent users of social media, who are able to navigate the complexity of the medium and its contents, and in relation to health and their bodies. At the same time, the adolescent girls sometimes found themselves, unintentionally, exposed to risk, particularly when social media was experienced publicly in a temporal order, connected to the past or present, and without a control of potential future effects and impacts. While the body has been traditionally considered a more private domain (Barker 1995), these girls were making bodies public and commenting on aspects of their own (and other’s) bodies, navigating in complex ways the thin boundary between public and private domains. In this way, they were constantly recreating the ‘new public’ (Gobetti 1992) and the ‘new private’. Judging what is appropriate vs inappropriate or interesting vs uninteresting for others thus include a competence to foresee how posted content can develop through responding feeds and comments. To comment wrongly about your own body can end up in fat talk, while at the same time to post the ‘wrong’ body can result in fat or skinny shaming.

While previous studies have shown the potential to specify cultural norms and power relations (e.g. body and gender) and investigate psychological variables (e.g. relationship between mental health and screen time) PEA, as illustrated in this paper, provides a tool to analyse body pedagogic processes (Shilling 2017, 2018), such as how embodied experience is structured during specific encounters with health-related social media. Furthermore, in the tradition of body pedagogics, empirical PEA results of how certain paradoxes are navigated can help us specify what is involved in the practical and cultural means through which adolescents both accommodate themselves to environmental conditions (consumer aspect) and adapt environmental conditions to their own needs (producer aspect) in a social media milieu. In this sense, adolescents uses of health-related social media is an example of an embodied knowledge that “not necessarily respect[s] divisions between social sectors or the public/private divide” (Shilling 2017, p. 1216), but instead develops through adolescents’ particular transactions with internal and external environments (Garrison 2001; Shilling 2008).

By acknowledging the adolescent girls’ social media competence and their risky navigation between private and public spaces, mechanisms of learning can here be explained as the girls’ creation of certain stories about their social life. Learning through stories was identified from the two indeterminate situations: (i) skinny fat and (ii) naturally fake, within the overarching paradox of publicly private. Within these situations, multiple gaps related to perceptions of the girls own bodies and others, and different variations of natural and fake bodies in relation to a ‘skinny’ ideal body shape and size were identified. In the data, we observed learning mechanisms in relation to stories of how the girls engaged with health, self, and gender via social media.

Stories of Self: The girls considered bodies (their own and others) as an ‘unfinished business’. This is also in accordance to previous literature that suggests that there has been a shift in the way that people consider bodies (Featherstone 1991). Previously, the body was considered as something that you were born with and that you could not change significantly (Leder 1990). Now, the body is ‘a project’ (Shilling 2016), something that you continuously and relentlessly should be working on to achieve prescribed ideals in relation to health, body shape and size (Coffey 2015; Gilleard 2002). In our study the girls’ actions, practices and comments supported this. At the same time these stories of self are not about ‘either you’re in or you’re out’, but about which stories to create and participate through in life on social media by being both producers and consumers of social media content. In so doing, the participants were constantly navigating between the boundaries of being critical, ambivalent and instrumental prosumers (Pang et al. 2019) of social media. In a sense social media becomes a space where they can express themselves and it relates both to accommodation and adaption of stories in their transactional engagement with social media.

Stories of Gender: In the data, there were notions of heternormativity and traditional ideals of female bodies. Even though there has been a shift in the ideals of a female body throughout the last few years from very slim to more toned-looking bodies (Boepple et al. 2016), the girls only made reference to these ideals of a female body, and did not include other possible body-shapes as an alternative ideal female body. In a sense it was a constant comparison of bodies in relation to a strong heterosexual norm of what beauty is, which highlights the impossibility of an in-between conception of the female body. Closely related to this quite narrow perception of gender and female body ideals, was their invisibility to matters related to ethnicity, and how notions of gender and body ideals are in close relation to ethnic background. This is an interesting finding given the sample and the engagement of girls in the research from different ethnicities.

Stories of Health: In the data, health was closely connected to a visual presentation of the body. The girls in general demonstrated a narrow view of what health is, and they considered ‘health = going to the gym and eating healthily’. This involves a story of progression of shaping the body and always trying to become better. This is in accordance with previous studies (e.g. Pang, Alfrey, and Varea 2016; Powell and Fitzpatrick 2015) which suggest that young people tend to have quite limited perceptions of health. Furthermore, and in alignment with previous literature (e.g. Markula, Burns, and Riley 2008; Varea and Pang 2018) the girls considered that ‘health is something visible’ and that they can judge if someone is healthy or not by the visual images they encounter on social media. In this sense, the appearance of the body becomes a principal determinant of health (Aphramor and Gingras 2008). At the same time, it was not only a ‘skinny’ ideal that was considered healthy. Normal, fit, ‘slim thick’ or ‘not too fake’ bodies were also related to health where a story of health does not seem to have validity unless it is connected to a path available to ‘normal people’. The girls spend much time navigating what should be considered ‘normal’, for example, what is fake and what is not fake. Through such micro-pedagogical practices they try to connect to a path available through social media health stories.

## Implications

The key challenge for adults, schools, policy makers or researchers is to resist dismissing, demonising, controlling or limiting young people’s social media practices that on the surface level appear harmful and representative of ridicule, insult and bullying. Instead, relevant adults should acknowledge young people’s vast competence of life on social media and further empower young people to self-regulate their learning through social media, as part of adolescence, and in ways that help them to learn from experiences about their health and bodies to shape future actions. A key action for adults who have a responsibility for young people is to facilitate health education in online and offline spaces. Schools and subjects like physical education and/or citizenship education (or equivalent, such as Personal Social and Health Education) need to be more proactive in shaping how diverse groups of young people make sense of health and their bodies through the stories they live by on and off social media. We accordingly conclude with some questions that have developed as a consequence of our study:

- How do we empower young people to use social media as an educative resource, and what are the key practices of schools to facilitate learning in, through and about health-related social media?
- How do young people learn to be safe users and producers of social media – and thus learn from the consequences of practices like body-shaming and fat talk?
- How should adults highlight the wealth of knowledge and competence that young girls have in regards to using social media in order to support young people?
- How do contemporary young people learn through stories and connect experiences between different stories and available pathways for action?

The stories of self, health and gender reported in this paper can act as a valuable starting point for helping relevant adults understand and engage with young people’s complex social media behaviours, and their health-related needs. As argued by Smith et al. (2013), stories are an effective knowledge translation tool because they enable research to be presented in a way that is more understandable, more humane and more memorable, where stories can open up rather than close down different ways of thinking. The key point to make is that research is potentially more productive and impactful when young people’s perspectives on social media engagement are generated, and when the findings reporting on the complexity of social media engagement are reported authentically, such as through the voices of young people and their stories.

## Notes

1. https://www.gov.uk/types-of-school.

2. Slim thick is a girl with a slim body shape but a big booty and big thighs. Usually with a small waist and flat stomach. www.urbandictionary.com.

3. For the girls in this paper, fat shaming was a practice of telling people that they shouldn’t be fat.

4. Stories are videos and images that appear outside if the regular social media feed and last for 24 hours before they disappear. They are temporary but can be organised into small collections for later viewing.
